# Long-term monitoring of mammal communities in the Peneda-Gerês National Park using camera-trap data

**DOI:** 10.3897/BDJ.11.e99588

**Published:** 2023-04-20

**Authors:** Annika M. Zuleger, Andrea Perino, Florian Wolf, Helen C. Wheeler, Henrique M. Pereira

**Affiliations:** 1 Institute of Biology, Martin Luther University Halle-Wittenberg, Halle (Saale), Germany Institute of Biology, Martin Luther University Halle-Wittenberg Halle (Saale) Germany; 2 German Centre for Integrative Biodiversity Research (iDiv) Halle-Jena- Leipzig, Leipzig, Germany German Centre for Integrative Biodiversity Research (iDiv) Halle-Jena- Leipzig Leipzig Germany; 3 School of Life Sciences, Anglia Ruskin University, Cambridge, United Kingdom School of Life Sciences, Anglia Ruskin University Cambridge United Kingdom; 4 BIOPOLIS Program in Genomics, Biodiversity and Land Planning, CIBIO, Vairão, Portugal BIOPOLIS Program in Genomics, Biodiversity and Land Planning, CIBIO Vairão Portugal

**Keywords:** camera traps, mammal, Portugal, long-term monitoring, occupancy, density

## Abstract

**Background:**

In the past decades, agricultural land abandonment and declining land-use intensity became common, especially in the Mediterranean countries of southern Europe. In some areas, this development opened up possibilities for rewilding and the recolonisation or expansion of large mammal populations. Yet, in some instances, co-occurrence of wild mammals and free-ranging domestic herbivores might lead to potential conflicts. It is, therefore, necessary to study the ecological interactions between wild and domestic mammal species to understand the effects of land abandonment and rewilding on biodiversity and ecosystem services. Camera traps are an effective tool for studying species interactions and occupancy dynamics as they allow for long-term monitoring with minimal interference. We conducted a long-term monitoring programme with camera traps in the Peneda-Gerês National Park in northern Portugal. The area has undergone substantial land-use changes following the abandonment of agricultural areas in the past 60 years. While agro-pastoral activities, especially the breeding of free-ranging horses and cattle, are still common in the area, the intensity of these activities has decreased significantly, promoting natural succession and an increase or return of several large mammal species in recent years. Overall, our project aims at: (1) assessing the population trends of the medium and large sized mammals in the area over time; (2) analysing the effects of passive rewilding on occurrence, abundance and behaviour; and (3) understanding potential interactions or conflicts between wild and domestic herbivores. In this publication, we present results of a primary occupancy analysis between 2015 and 2020, as well as a comparison between occupancy and density estimates for 2019.

**New information:**

Our publication provides a dataset from long-term camera-trap monitoring in the Peneda-Gerês National Park between 2015 and 2021. We established a 16 km² grid of 64 cameras deployed yearly during the summer months. Together with this publication, we publish the data and images collected between 2015 and 2021, using both the Camtrap DP standard and the GBIF Darwin Event Core. We obtained a total of 934,810 pictures on 41,234 trap nights. The pictures were automatically grouped into sequences with each sequence representing a distinct occurrence event, resulting in 80,191 occurrences. Out of those, 14,442 contained observations of a species, while the remaining were either blank or the species was not identifiable. We only obtained the information whether a species was present or absent on a picture, disregarding the number of individuals. Most observations were of domestic cattle (*Bostaurus*) and horses (*Equuscaballus*), followed by European roe deer (*Capreoluscapreolus*) and wild boar *(Susscrofa*). Further observations include red fox (*Vulpesvulpes*), gray wolf (*Canislupus*), Eurasian badger (*Melesmeles*), stone marten (*Martesfoina*), common genet (*Genettagenetta*), Iberian ibex (*Caprapyrenaica*) and red deer (*Cervuselaphus*). We estimated occupancy and densities for the most common species. The project is on-going and additional data will be included in the future. The dataset is freely available for ecological analysis, but also for training machine-learning systems in automated image classification as all pictures have been manually classified.

## Introduction

Agricultural land abandonment and decreasing land-use intensity have been an increasingly important issue in Europe especially in Mediterranean countries, such as Spain (Gabarrón-Galeote et al. 2015) and Portugal (Nunes et al. 2010). Between 1990 and 2006, a total area of almost 120,000 ha was affected by abandonment processes in southern Europe (Hatna and Bakker 2011). This land abandonment is often associated with significant changes in biodiversity and ecosystem services. It can, for example, open up possibilites for reforestation or rewilding and the recolonisation or growth of large mammal communities (Navarro and Pereira 2015). This might lead to potential conflicts with low-intensity agricultural practices, such as the breeding of free-ranging domestic herbivores. Understanding the long-term impacts of the de-intensification or abandonment of agricultural areas on wild mammal populations, as well as the potential interactions with domestic herbivore species in the area is, therefore, important for evaluating the effects of abandonment on biodiversity and ecosystem services. The Peneda-Gerês National Park in northern Portugal underwent such land-use changes following a rural exodus and abandonment of agricultural areas since the 1950s. This promoted a natural regeneration of oak forests, an increase in some local mammal populations and opened possibilities for passive rewilding of the area. For example, in 1997, the Iberian ibex (*Caprapyrenaica*) was re-introduced in Galicia, Spain close to the Portuguese border and is, since then, recolonising the area (Fonseca et al. 2017). Yet, the breeding of free-ranging cattle and horses is still a common agricultural practice. This creates opportunities to study and monitor the effects of domesticated herbivores on the occurrence, density and behaviour of local wild mammal species and vice versa.

Camera traps have proven to be an effective tool for this monitoring as they allow researchers to collect data while limiting interference with animals. They can provide information on distribution, behaviour, species richness or population dynamics (Rowcliffe and Carbone 2008; O’Connell et al. 2011; Burton et al. 2015; Caravaggi et al. 2017). Moreover, they improve the monitoring of elusive species that tend to avoid human observers and increase comparability and replicability as observations are stored as visual information. The information obtained can be used for a great spectrum of different ecological analyses ranging from occupancy modelling (MacKenzie et al. 2002) over the estimation of abundances of marked (e.g. capture-recapture, Karanth (1995)) and even unmarked animals (see Gilbert et al. (2020) for a review of recently developed methods).

We developed a long-term wildlife monitoring programme in the Peneda-Gerês National Park using camera traps. The programme was initiated in 2015 and, since then, camera traps are deployed every year during the summer months (April - October). The aim is to analyse temporal trends throughout the study duration. For this study, we were interested in obtaining occupancy as well as density estimates for the species observed and comparing the two metrics. Occupancy is a frequently-used metric in monitoring programmes to understand how a species is distributed in space because it can be obtained from presence-absence data of even unmarked individuals. Yet, the density or abundance of species contains a higher informational value regarding the viability of a population and is directly comparable through time and space. We applied both occupancy modelling following MacKenzie et al. (2002), as well as camera trap distance sampling (CTDS) following Howe et al. (2017).

## General description

### Purpose

The long-term monitoring project aims to: (1) assess the population trends of the medium- and large-size mammals of the region over time; (2) analyse the effects of passive rewilding and other environmental variables on their occurrence and abundance; (3) look at potential interactions between wild and domestic species and (4) analyse effects of environmental and anthropogenic variables on their behaviour (e.g. activity patterns). In this publication, we focus on analysing occupancy trends of the observed species between 2015 and 2020, as well as comparing occupancy estimates with density estimates obtained in 2019.

## Project description

### Study area description

**Figure 1.**
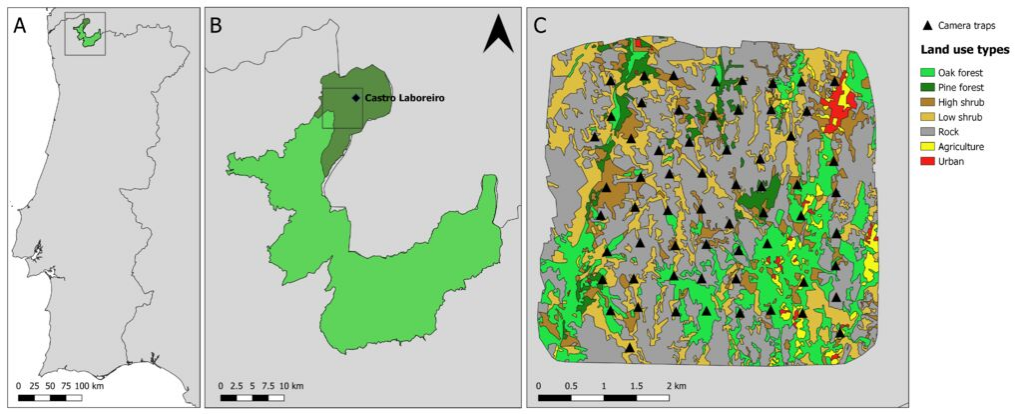
A) Location of the Peneda-Gerês National Park. B) Location of the Castro Laboreiro and Lamas de Mouro parishes. C) Camera-trap locations and land-use types within the survey area. Cameras were placed randomly with regard to animal density and activity, but the locations were chosen in a way to represent the different land-use types in the area relative to their overall occurrence.

The study was conducted in the parishes of Castro Laboreiro and Lamas de Mouro in the Peneda-Gerês National Park in northern Portugal (Fig. 1). The Park was created in 1971 with the aim to protect the high natural value landscape. It is the oldest protected area and the only national park in Portugal. Over the past 60 years, the region has undergone substantial land-use changes (van der Zanden et al. 2018). These changes were especially marked in the area surrounding Castro Laboreiro, where historically, agro-pastoral activities were common, with seasonal migration from summer villages at the plateau to winter villages in the valley (van der Zanden et al. 2018) and socio-economic changes had led to a large population decline (Rodrigues 2010). This abandonment opened possibilities for natural succession and passive rewilding in the area. Today, the area supports a diversity of habitats consisting of small agricultural fields in the valley, shrublands and oak forest patches in the hillside and pastures for cattle and agricultural fields in the plateau (Rodrigues 2010). Since 1971, Castro Laboreiro is part of the Peneda-Gerês National Park and since 1997, it is also part of the European protected area network Natura 2000.

The elevation in the area ranges from 300 to 1,340 m (van der Zanden et al. 2018). As it is located at the transition between the Mediterranean and Atlantic biogeographic zones, the region has a temperate Mediterranean climate, characterised by cold and rainy winters and warm summers (van der Zanden et al. 2018). The average annual temperature throughout the study duration was 11.9°C at 800 m elevation, with an average annual precipitation of 1858 mm between 1985 and 2015 (SNIRH - Sistema Nacional de Informação de Recursos Hídricos 2022). Within the camera-trap grid, we identified seven different land-use types from aerial imagery in 2020, with bare rock accounting for almost half of the area (45.30%) followed by low shrub (20.13%) and oak forest (18.77%). While agriculture (2.42%) and urban infrastructure (1.11%) only represent a considerably small part of the area, high shrub and pine forest make up for 8.64% and 3.63%, respectively (Fig. 1).

## Sampling methods

### Study extent

The dataset is obtained from a long-term monitoring campaign that has been conducted every year since 2015. Currently, images from 2015 to 2021 are classified, but further data will be included in the future. In 2015 and 2016, camera traps were deployed from April to August and from 2017 to 2019 from May to October (see Table 1 for details). In 2020, because of travel restrictions during the Covid-19 pandemic, camera traps could only be deployed in June and were left in the field until May 2021. Due to theft and malfunction, the number of operative cameras ranged from 61 in 2016 to 48 in 2020/21. Trap-nights per year ranged from 4,236 in 2015 to 9,606 in 2020/21.

### Sampling description

For the study, 64 camera traps (Reconyx Hyperfire HC600, Holmen, WI, USA) were deployed in a 16 km² grid southwest of Castro Laboreiro. They were distributed as uniformly as possible across the different land-use types (e.g. 10% of cameras in land-use types that cover 10% of the area) with one camera per 0.25 ha grid cell (approx. 500 m spacing between each camera, Fig. 1). Real locations could deviate by up to 100 metres from the planned locations due to accessibility and placement possibilities. Further, some locations had to be adjusted throughout the years because of changes in the vegetation structure or due to theft, but new locations were within 100 m of the original location and placed in similar habitats. The coordinates in the dataset were rounded to three decimals to protect the camera traps from theft.

The cameras remained active for 24 hours per day and were programmed on motion sensor to take three consecutive pictures each time they were triggered by an animal with no delay after a trigger event. The sensitivity of the sensor was set to high in 2015, 2016 and 2019, medium in 2017 and 2018 and medium/high in 2020 (see eventRemarks). Sampling effort was measured as the number of camera traps multiplied by the number of days they remained active (Rovero et al. 2010).

### Quality control

To ensure using the updated scientific name and common name of species, the taxonomic nomenclature followed the Catalogue of Life (https://www.catalogueoflife.org). Additionally, we checked every species in the database of the IUCN Red List of Threatened species (https://www.iucnredlist.org) for their conservation status and populations trends.

### Step description

Each image obtained from the camera traps was classified manually. The images were later imported into Agouti (Casaer et al. 2019, https://www.agouti.eu) to be made publicly available. There they were automatically grouped into sequences of contiguous images with each sequence representing one distinct occurrence event. The pre-classified observations were linked to the respective sequences using the image name. Starting with the data from 2020, the images were directly imported into Agouti and classified within the software. The dataset was exported from Agouti in Camtrap DP format (Camtrap DP Development Team 2021) and manually converted to Darwin Core standard (Darwin Core Task Group 2009). It is structured as a sample event dataset including the event and the occurrence data and published as a Darwin Core Archive (DwC-A). Here, an event refers to a camera-trap deployment at a certain location over a certain amount of time (equivalent to *deployment* in Camtrap DP format) and an occurrence refers to a distinct occurrence event (here: sequence, equivalent to *observation* in Camtrap DP format). The published DwC-A dataset only includes occurrences that contained observations of an animal. As the DwC-A format currently does not allow for hierarchical datasets with more than two levels, we included the first ten images of each occurrence event as associatedMedia in the occurrence table. The original Camtrap DP dataset including also blank and unknown occurrences will be published as supplemental material to this publication (Suppl. material 4, Suppl. material 5) and made available through GBIF in the future. The media file containing information and identifiers for all images will be published once the Camtrap DP integration in GBIF is possible and is, until then, available upon request.

## Geographic coverage

### Description

Castro Laboreiro and Lamas de Mouro, Peneda-Gerês National Park, Portugal.

### Coordinates

41.997 and 42.036 Latitude; -8.204 and -8.158 Longitude.

## Taxonomic coverage

### Description

Mammals and birds were identified to the species level where possible.

### Taxa included

**Table:** 

Rank	Scientific Name	Common Name
class	Mammalia	Mammals
class	Aves	Birds

## Temporal coverage

**Data range:** 2015-4-19 – 2015-8-19; 2016-4-13 – 2016-8-27; 2017-5-08 – 2017-10-03; 2018-5-15 – 2018-10-15; 2019-5-07 – 2019-10-08; 2020-6-02 – 2021-5-07.

## Usage licence

### Usage licence

Creative Commons Public Domain Waiver (CC-Zero)

## Data resources

### Data package title

Long-term monitoring of mammal communities in the Peneda-Gerês National Park using camera-trap data

### Number of data sets

2

### Data set 1.

#### Data set name

CT_Peneda_events

#### Data format

Darwin Core Archive format

#### Download URL

https://doi.org/10.15468/rah33j

#### Data format version

Version 1

#### Description

The dataset is published in the Global Biodiversity Information Facility platform, GBIF (Zuleger et al. 2023). It includes all observations of a species where classification was possible. Observations of humans are excluded from the dataset. It is structured as a sample event dataset that has been published as a Darwin Core Archive (DwC-A), which is a standardised format for sharing biodiversity data as a set of one or more data tables. This data file contains the 331 camera-trap deployments during a certain year (eventID) from which camera-trap images were obtained. The GBIF IPT (Integrated Publishing Toolkit, Version 2.5.6-rd6f172f) archives the data and thus serves as the data repository. The data and resource metadata are available for download from GBIF. Additionally, the original Camtrap DP dataset including deployment and observation data is made available as supplemental data to this publication. The media data will be made available through GBIF once the integration of the Camtrap DP format is possible and is, until then, available upon request.

**Data set 1.** 

Column label	Column description
eventID	Unique identifier for the set of information associated with the event.
locationID	Identifier for the location information, here: Camera-trap location in a certain year.
decimalLatitude	Geographic latitude (in decimal degrees).
decimalLongitude	Geographic longitude (in decimal degrees).
geodeticDatum	Geodetic datum or spatial reference system (SRS) upon which the geographic coordinates given in decimalLatitude and decimalLongitude are based, here: WGS84.
coordinateUncertaintyInMetres	The horizontal distance (in metres) from the given decimalLatitude and decimalLongitude describing the smallest circle containing the whole of the Location, here: 100 m as coordinates were rounded to three decimals.
verbatimCoordinates	Verbatim original spatial coordinates of the Location.
verbatimCoordinateSystem	Coordinate format for the verbatimCoordinates of the Location, here: decimal degrees.
verbatimSRS	The ellipsoid, geodetic datum or spatial reference system (SRS) upon which coordinates given in verbatimLatitude and verbatimLongitude, or verbatimCoordinates are based, here: WGS84.
eventDate	Interval during which an Event occurred, here: time camera was deployed and functional.
higherGeography	List of geographic names less specific than the information captured in the locality term.
continent	Name of the continent in which the Location occurs, here: Europe.
country	Name of the country in which the Location occurs, here: Portugal.
countryCode	Standard code for the country in which the Location occurs, here: PT.
stateProvince	Name of the next smaller administrative region than country (state, province, canton, department, region etc.) in which the Location occurs, here: Norte.
county	Full, unabbreviated name of the next smaller administrative region than province (county, shire, department etc.) in which the Location occurs, here: Viana do Castelo.
municipality	Full, unabbreviated name of the next smaller administrative region than county (city, municipality etc.) in which the Location occurs.
locality	Specific description of the place.
verbatimElevation	Elevation (above sea level) of the Location in metres.
ownerInstitutionCode	Name of the institution having ownership of the information referred to in the record, here: iDiv.
samplingProtocol	Protocol used during an Event, here: camera traps.
samplingEffort	The amount of effort expended during an Event.
sampleSizeValue	Numeric value for a measurement of the size of a sample in a sampling event, here: number of trap nights.
samplieSizeUnit	Unit of measurement of the size of a sample in a sampling event, here: trap-nights.
eventRemarks	Additonal remarks regarding the setting of the camera traps, here: Trigger Sensitivity setting of the camera trap.

### Data set 2.

#### Data set name

CT_Peneda_occurrences

#### Data format

Darwin Core Archive format

#### Data format version

Version 1

#### Description

The dataset is published in the Global Biodiversity Information Facility platform, GBIF (Zuleger et al. 2023). It includes all observations of a species where classification was possible. Observations of humans are excluded from the dataset. It is structured as a sample event dataset that has been published as a Darwin Core Archive (DwC-A), which is a standardised format for sharing biodiversity data as a set of one or more data tables. This data file contains the 14,509 sequences (occurrenceID) on which a species was observed (occurrenceStatus = present). It is linked to the camera trap deployments through the eventID. The dataset also includes the information on the respective species as well as the first ten images associated with this occurrence. The data and resource metadata are available for download from GBIF. Additionally, the original Camtrap DP dataset including deployment and observation data (including also blank sequences) is made available as supplemental data to this publication. The media data will be made available through GBIF once the integration of the Camtrap DP format is possible and is, until then, available upon request.

**Data set 2.** 

Column label	Column description
eventID	Unique identifier for the set of information associated with the event.
occurrenceID	Unique identifier for an occurrence, here: sequence of images referring to a distinct occurrence event.
basisOfRecord	The specific nature of the data record, here: MachineObservation.
occurrenceStatus	A statement about the presence or absence of a Taxon at a Location, here only presences are included.
eventDate	Date when the event was recorded.
year	The four-digit year in which the Event occurred.
month	The integer month in which the Event occurred.
day	The integer day of the month on which the Event occurred.
eventTime	Time at which the event occurred.
samplingProtocol	Description of the methods or protocols used during an event, here: camera trap.
occurrenceRemarks	Comments or notes about the Occurrence, here: captureMethod: motion detection.
taxonID	Identifier for the set of taxon information (data associated with the Taxon class).
identificationRemarks	Comments or notes about the Identification, here: classificationMethod: human or machine.
identifiedBy	Person, group or organisation who assigned the Taxon to the subject.
organismName	Textual name or label assigned to an Organism instance.
scientificName	Full scientific name in lowest level taxonomic rank that can be determined.
higherClassification	A list (concatenated and separated) of taxa names terminating at the rank immediately superior to the taxon referenced in the taxon record.
kingdom	Full scientific name of the kingdom in which the taxon is classified.
phylum	Full scientific name of the phylum or division in which the taxon is classified.
class	Full scientific name of the class in which the taxon is classified.
order	Full scientific name of the order in which the taxon is classified.
family	Full scientific name of the family in which the taxon is classified.
genus	Full scientific name of the genus in which the taxon is classified.
specificEpithet	Name of the first or species epithet of the scientificName.
infraspecificEpithet	Name of the lowest or terminal infraspecific epithet of the scientificName, excluding any rank designation.
taxonRank	Taxonomic rank of the most specific name in the scientificName.
associatedMedia	A list (concatenated and separated) of identifiers (URL) of media associated with the occurrence, here: the first 10 images of an occurrence (if more than 10 images were obtained). All images can be obtained from the Camtrap DP media file, which will be published through GBIF once the integration of the Camtrap DP format is available and is, until then, available upon request.

## Additional information

### Analysis

The purpose of this study was to obtain an estimate of naïve occupancy and abundance while accounting for imperfect detection and without relying on individual identification of the animals. For occupancy, we followed the modelling approach of MacKenzie et al. (2002). It assumes that, if a species is detected at a location, it can be considered present. Yet, non-detection of a species does not equal absence as the species might have gone undetected by the observer. The model, therefore, uses repeat sampling and the obtained detections and non-detections at each site on each survey to estimate the expected occupancy probability (\begin{varwidth}{50in}\begin{equation*}
            \psi
        \end{equation*}\end{varwidth}) and the probability of detecting the species given that it is present (\begin{varwidth}{50in}\begin{equation*}
            p
        \end{equation*}\end{varwidth}). In doing so, it allows the estimation of the probability of site occupancy when the detection probabilities are <1.

We fitted a single-season occupancy model for each year separately using a Maximum Likelihood framework and calculated the Maximum Likelihood for \begin{varwidth}{50in}\begin{equation*}
            \psi
        \end{equation*}\end{varwidth} and \begin{varwidth}{50in}\begin{equation*}
            p
        \end{equation*}\end{varwidth} following MacKenzie et al. (2002) by:

\begin{varwidth}{50in}\begin{equation*}
            L\left(\psi,p\right)=\left[\psi^{n.}\prod_{t=1}^{T}p_t^{n_t}\left(1-p_t\right)^{n-n_t}\right]\ast\left[\psi\prod_{t=1}^{T}\left(1-p_t\right)+\left(1-\psi\right)\right]^{N-n.} 
        \end{equation*}\end{varwidth} Eq. 1

with time (\begin{varwidth}{50in}\begin{equation*}
            t
        \end{equation*}\end{varwidth}), the total number of surveyed sites (\begin{varwidth}{50in}\begin{equation*}
            N
        \end{equation*}\end{varwidth}), the number of distinct sampling occasions (\begin{varwidth}{50in}\begin{equation*}
            T
        \end{equation*}\end{varwidth}), the number of sites where the species was detected at time \begin{varwidth}{50in}\begin{equation*}
            t
        \end{equation*}\end{varwidth} (\begin{varwidth}{50in}\begin{equation*}
            n_t
        \end{equation*}\end{varwidth}) and the total number of sites at which the species was detected at least once (\begin{varwidth}{50in}\begin{equation*}
            n.
        \end{equation*}\end{varwidth}). The detection matrices of presence/absence records used for analysis consisted of one record per species, camera location and day. We used the R package *unmarked* (Fiske and Chandler 2011) to estimate occupancy and detection probabilities, as well as standard errors (SE). R code for the occupancy analysis is available in Suppl. material 6.

For the estimation of population densities, we applied the camera-trap distance sampling (CTDS) methodology developed by Howe et al. (2017) to the data from 2019. CTDS applies the distance sampling framework by Buckland et al. (2004) without the need for individual identification and allows us to account for imperfect detection. Here, a camera trap which is deployed at point \begin{varwidth}{50in}\begin{equation*}
            k
        \end{equation*}\end{varwidth} for a period of time \begin{varwidth}{50in}\begin{equation*}
            T_k
        \end{equation*}\end{varwidth} serves as the observer. Density can be estimated as:

\begin{varwidth}{50in}\begin{equation*}
            \hat D = \frac{{2t\sum\limits_{k = 1}^K {{n_k}} }}{{\theta {w^2}\sum\limits_{k = 1}^K {{T_k}{{\hat P}_k}} }}
        \end{equation*}\end{varwidth} Eq. 2

where *ϴ* is the horizontal angle of view of the camera model, \begin{varwidth}{50in}\begin{equation*}
            w
        \end{equation*}\end{varwidth} is the truncation distance beyond which observations are discarded, \begin{varwidth}{50in}\begin{equation*}
            n_k
        \end{equation*}\end{varwidth} is the number of animals recorded at point \begin{varwidth}{50in}\begin{equation*}
            k
        \end{equation*}\end{varwidth} and \begin{varwidth}{50in}\begin{equation*}
            \hat{P}_k
        \end{equation*}\end{varwidth} is the probability at each snapshot moment that an animal within the survey area is detected between 0 and \begin{varwidth}{50in}\begin{equation*}
            w
        \end{equation*}\end{varwidth} in front of the camera (Howe et al. 2017).

We measured the distance to the camera of each animal present on a picture by visually comparing them to reference distances recorded during the deployment in that year. A detection function was fitted to the observation distances in the software Distance 7.3 (Thomas et al. 2010) using a conventional distance sampling approach with a point transect model. Two different key functions (Hazard-rate and Half-normal) with up to two cosine adjustment terms were applied and non-parametric bootstrapping with 999 iterations generated by sampling with replacement from the obtained data being used to calculate the coefficient of variation (Buckland et al. 2004). We estimated \begin{varwidth}{50in}\begin{equation*}
            \hat{P}
        \end{equation*}\end{varwidth} as the mean detection probability of all models weighted by their AIC (Akaike Information Criterion; Akaike (1973)). The density estimates were corrected for the limited availability of species (Howe et al. 2017) by estimating an availability factor \begin{varwidth}{50in}\begin{equation*}
            a
        \end{equation*}\end{varwidth} which was derived by calculating the proportion of time animals spend active (activity level), following Rowcliffe et al. (2014).

Additionally, we calculated the mean encounter rate \begin{varwidth}{50in}\begin{equation*}
            \bar{\varepsilon}
        \end{equation*}\end{varwidth} (number of observations per camera) across all cameras and the Coefficient of Variation (CV) of the density estimates following the delta method (Dorfman 1938):

\begin{varwidth}{50in}\begin{equation*}
            CV\left(\hat{D}\right)^2=CV\left(\bar{\varepsilon}\ \right)^2+CV\left(\hat{p}\right)^2+CV\left(a\right)^2
        \end{equation*}\end{varwidth} Eq. 3

The CV of the encounter rate was obtained following the approach of Fewster et al. (2008) for estimating the encounter rate variance in distance sampling. The CV of the detection probability was calculated as the CV of all models weighted by their AIC. The CV of the activity level was calculated using bootstrapping with 999 iterations resampling from the original data (Rowcliffe et al. 2014).

### Results

We collected a total of 934,810 pictures on 41,234 trap days with the majority of the pictures being collected in 2015 and 2016. In 2017 and 2018, we obtained considerably less images and observations probably due to the lower sensitivity setting of the camera traps (Table 1). Grouping the pictures into sequences led to a total of 80,191 distinct occurrence events with a mean of 13.2 pictures per event. Out of those, 14,442 contained observations of an animal species. In total, we observed ten wild mammal species and six domestic species (Table 2). The most frequently observed species were domestic cattle (4,572) and domestic horses (4,554), followed by European roe deer (2,548) and wild boar (1,891).

**Figure 2.**
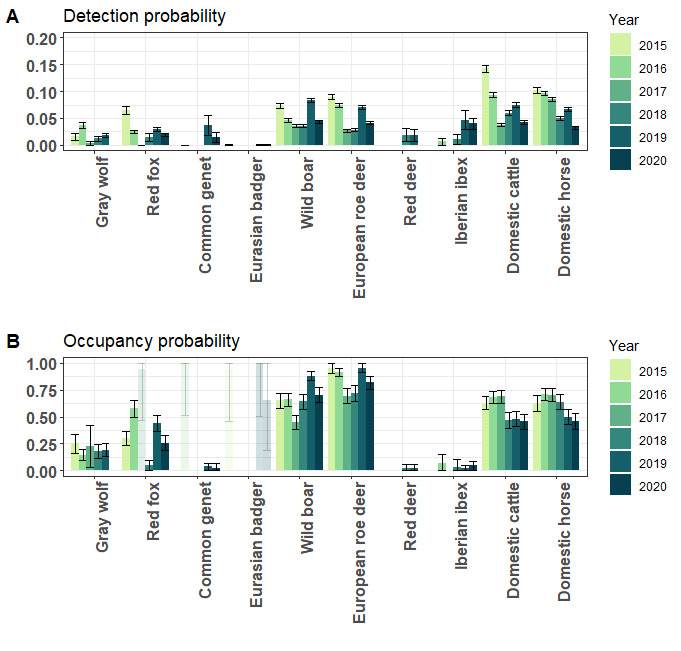
A) Detection (**p**) and B) occupancy (**Ψ**) probabilities for every species per year (see Suppl. material 1 for all values). Transparent bars mark models with too few observations to obtain reasonable results.

Detection probabilities obtained from occupancy modelling were generally low across species and approached zero for species with fewest observations (Fig. 2). The highest occupancy estimates throughout the study duration were obtained for European roe deer (0.72 - 1) and wild boar (0.48 - 0.92) (Fig. 2), yet their densities were considerably low (Table 3). In contrast to that, domestic cattle and horses showed very high density estimates, while their occupancy was rather low (0.47 - 0.72 and 0.46 - 0.73, respectively). It seems that the domestic species are slightly decreasing in occupancy over the years, while the wild species remain mostly constant. For all other species, we obtained comparatively low occupancy estimates over the years, but for many of them, the number of observations is too low to make any assumptions about temporal trends at this point (Table 2). In 2017, 2018 and 2020, most of the species showed lower occupancy estimates than in other years. A Spearman rank correlation analysis revealed that, for the more common species (e.g. cattle, horses, roe deer and wild boar), detection probabilities were positively correlated with the sensitivity setting of the cameras in that year (R = 0.5, p = 0.002). Yet, we found no significant correlation between occupancy and sensitivity (R = 0.11, p = 0.55). All occupancy and detection probabilities are presented in Suppl. material 1.

When looking at the density analysis, the obtained distances (Suppl. material 2) showed that many cameras only triggered up to 10 metres, even though the motion sensor range was reported to be up to 30 metres (manufacturer's information). For all species, observation distances decreased with increasing distance. Yet, for foxes and wild boar, we obtained very few observations close to the camera, indicating that some animals might have gone undetected at short distances. For both red fox and gray wolf, the density estimates were low with confidence intervals overlapping zero. Therefore, the results should be observed with care. Information and results of each model contributing to the final estimates are presented in Suppl. material 3.

**Figure 3.**
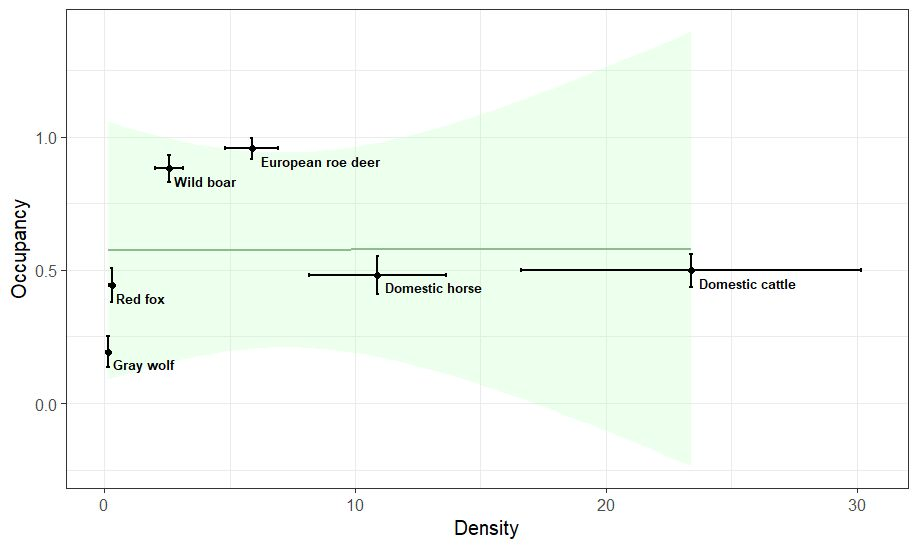
Comparison between occupancy estimates obtained from occupancy modelling (MacKenzie et al. 2002) and density estimates obtained through camera-trap distance sampling (Howe et al. 2017) for the six most common species in the study area in 2019.

Comparing the occupancy and the density estimates from 2019, we did not observe a strong relationship between them when including all six species in the analysis (R = 0.54, p = 0.30). Yet when only including the wild species, there is a very strong and marginally significant positive correlation between occupancy and density (R = 1, p = 0.08, Fig. 3). With higher densities, the occupancy probability and, therefore, the amount of space used by the population increases.

### Discussion

We are presenting an extensive dataset obtained from long-term camera trapping that will be maintained and extended in the future. The dataset will offer the opportunity to study local changes in occupancy and density, as well as responses to changes in land-use and vegetation structure or anthropogenic influences on a diverse mammal community. It is currently published in the Darwin Core Archive (DwC-A) format, but GBIF is in the process of implementing a new data model allowing for improved presentation of camera-trap data, as well as the upload of Camtrap DP data in the Integrated Publishing Toolkit (IPT). As the DwC-A standard is not very well suited for camera trap data, for example, by not allowing the hierarchical structure of deployments, media and observations and the manual conversion from Camtrap DP to DwC-A has proven to be challenging (e.g. it was only possible to include images as associatedMedia in the occurrences and not as a distinct source data), we will adopt the new data model once available. Our dataset can then be used for the training for automated computer classification systems, as all images will be made available online.

The very simple index of occupancy presented in this paper already shows that there might be certain trends, such as a decrease in the space used by domestic species and a slightly increasing occupancy of species, such as the red deer or the Iberian ibex that are currently in the process of repopulating the area (Moço et al. 2006; Santos et al. 2011). The data from following years will hopefully render further insights into those processes, especially if also environmental variables or multiple species interactions are included in the analysis. However, we observed an effect of the sensitivity setting of the camera traps on the detection probabilities which needs to be explored in more depth in future analysis by incorporating it directly in modelling. Additionally, it needs to be considered that, because of the design of our camera trapping grid (cameras being only 500 m apart), the assumption of independence amongst sampling locations (MacKenzie et al. 2002) is very likely to be violated, so \begin{varwidth}{50in}\begin{equation*}
            \psi
        \end{equation*}\end{varwidth} should be interpreted with care.

Regarding the density estimates obtained from CTDS, our results were consistent with literature from the same or similar study areas. Unfortunately, for many species, there is no recent literature on population densities. For example, for the gray wolf the last national assessment of populations in Portugal was performed in 2002 - 2003 by Pimenta et al. (2005) using a combination of different indirect sampling methods and resulting in a much lower density estimate of 0.03 – 0.06 individuals/km² (compared to 0.15 individuals/km² in our study) for the study area. Yet, several studies have recorded an increase in wolf numbers and positive population growth rates in Portugal since 2007 (Campos 2018). For roe deer, a relatively recent study by Valente et al. (2014) estimated densities using the distance-sampling framework with data from pellet-group counts in the Montesinho Natural Park (approximately 100 km east of the survey area) and calculated a density of 3.5 (2.26 – 5.45) individuals per km² (5.88 individuals/km² in our study). Many roe deer populations have increased greatly in Portugal within the last 20 years (Torres et al. 2015), so an increase in the Peneda-Gerês National Park due to changes in land-use practices and re-naturalisation of habitats is likely. To date, there are no studies estimating wild boar abundances or densities in northern Portugal. Bosch et al. (2012) and Pittiglio et al. (2018) have conducted surveys using habitat suitability models to predict wild boar densities in northern Portugal. Yet, their estimates were close to zero. This shows that modelled densities from remote sensing data, based on assumptions about habitat suitability, should always be interpreted with care. Data from the National Statistics Institute (Instituto Nacional de Estatística (INE) 2019) reported cattle densities in the study area to be 18.76 individuals/km², ranging from 9 individuals/km² in the Lamas de Mouro and Castro Laboreiro area to 37 individuals/km² in the Gaveira Region (Instituto Nacional de Estatística (INE) 2019). Our estimates (approx. 23 individuals/km²) lie within this interval. For horses, we also obtained data from the INE. Yet, these data seem to be underestimated as it only reports a density of 0.76 individuals/km² and about 115 horses for the region, whereas we obtained densities of almost 11 individuals/km².

For future analysis, we aim at analysing temporal trends, not only in occupancy, but also in abundance and investigate the occupancy-abundance-relationship in more depth. With the data from just one year, it was not possible to observe a relationship between those two metrics. Potential reasons are that we only have one estimate per species for the entire study area and the relationship might depend on species specific variables, such as home range or site specific variables, such as habitat (Efford and Dawson 2012). Another reason is that there can be time lags in the response of occupancy to increases and decreases in abundance that only become apparent from long-term monitoring initiatives (Gaston et al. 2000). Yet, if only considering the wild species, we do observe that higher populations densities could be correlated with higher occupancy estimates. While the domestic species seem to appear in larger groups only in certain parts of the study area, leading to high density estimates with lower occupancy estimates, wild species seem to have higher densities with increasing occupancy. We expect that new technological advancements will soon make the application of CTDS to previously-collected data possible, so that we are planning to also obtain density estimates for the years of 2015 - 2018 and from 2020 onwards. This should hopefully give us further insights into the relationship of occupancy and abundance. Additionally, to including data from several years, we plan to also include environmental variables, as well as potential interactions between wild and domestic species in the analysis, to gain a better understanding of the relationship on a finer spatial scale.

## Supplementary Material

DD6FA1E4-45E5-5EA3-8F70-F3101BB2E8C9 10.3897/BDJ.11.e99588.suppl1 Supplementary material 1 Occupancy and detection probabilities per species and year Data type Table (.pdf) Brief description All occupancy and detection probability estimates for each species per year including standard error (SE). File: oo_789811.pdf https://binary.pensoft.net/file/789811 Annika M. Zuleger

19BD6855-2D0C-5608-9E9B-F3D5B2A07AE2 10.3897/BDJ.11.e99588.suppl2 Supplementary material 2 Observation distances Data type Figure (.jpg) Brief description Observed distances of the six most common mammal species used for the distance-sampling models. File: oo_770895.jpg https://binary.pensoft.net/file/770895 Annika M. Zuleger

B5874D79-8B78-5AF9-96AE-10BF6A9F96EF 10.3897/BDJ.11.e99588.suppl3 Supplementary material 3 Distance-sampling models Data type Table (.pdf) Brief description All models that were fitted with Distance 7.3 and weighted by AIC across the total survey area. File: oo_789814.pdf https://binary.pensoft.net/file/789814 Annika M. Zuleger

2848ADB7-2C3A-5104-B257-7F0E9AA7F708 10.3897/BDJ.11.e99588.suppl4 Supplementary material 4 CamtrapDP_deployments_Peneda Data type deployments (.csv) Brief description Camera trap deployments in Camtrap DP format.
deploymentID - Unique identifier of the deployment.
locationID - Unique identifier of the deployment location.
locationName - Name given to the deployment location.
longitude - Longitude of the deployment location in decimal degrees, using the WGS84 datum.
latitude - Latitude of the deployment location in decimal degrees, using the WGS84 datum.
start - Date and time at which the deployment was started. Formatted as an ISO 8601 string with timezone designator.
end - Date and time at which the deployment was ended. Formatted as an ISO 8601 string with timezone designator. File: oo_789819.csv https://binary.pensoft.net/file/789819 Annika M. Zuleger

8048F2FB-5ED0-51AE-80DE-8BFC61BFEBB4 10.3897/BDJ.11.e99588.suppl5 Supplementary material 5 CamtrapDP_observations_Peneda Data type observations (.csv) Brief description Observations in Camtrap DP format.
observationID - Unique identifier of the observation.
deploymentID - Unique identifier of the deployment the observation belongs to.
sequenceID - Unique identifier of the sequence (collection of media files grouped by a predefined `package.project.sequenceInterval`) that is the source of the observation.
timestamp - Date and time of the observation. Formatted as an ISO 8601 string with timezone designator (`YYYY-MM-DDThh:mm:ssZ` or `YYYY-MM-DDThh:mm:ss±hh:mm`). For file-based observations, this is the `media.timestamp` of the associated media file (in `mediaID`), for sequence-based observations the `media.timestamp` of the first media file in the associated sequence (in `sequenceID`).
observationType - Type of the observation.cameraSetup`true` if the observation is part of the camera setup process (camera deployment, pickup, maintenance).
taxonID - Unique identifier of the `scientificName` as defined in `package.taxonomic.taxonID` for that scientific name.scientificNameScientific name of the observed individual(s).
classificationMethod - Classification method.
classifiedBy - Name or unique identifier of the person or AI algorithm that classified the observation.
classificationTimestamp - Date and time of the classification. Formatted as an ISO 8601 string with timezone designator.
classificationConfidence - Accuracy confidence of the classification. Expressed as a probability, with `1` being maximum confidence. File: oo_789818.csv https://binary.pensoft.net/file/789818 Annika M. Zuleger

672C9B15-E4A8-545C-AA8C-64F4B8C177A5 10.3897/BDJ.11.e99588.suppl6 Supplementary material 6 R Script Long-term monitoring - Occupancy analysis Data type R Code Brief description R Code for the occupancy analysis presented in this publication. File: oo_819174.R https://binary.pensoft.net/file/819174 Annika M. Zuleger

## Figures and Tables

**Table 1.** Summary statistics of all years. *observations of humans are excluded.

**Year**	**No. Cameras**	**Deployed**	**Ended**	**Trap-days**	**Pictures**	Sequences total*	**Sequences observation**
**2015**	58	19.04.2015	19.08.2015	4,236	295,562	16,928	2,850
**2016**	61	13.04.2016	27.08.2016	6,744	280,239	18,129	3,761
**2017**	54	08.05.2017	03.10.2017	7,169	52,542	4,608	1,715
**2018**	58	15.05.2018	15.10.2018	6,649	31,437	11,238	1,283
**2019**	57	07.05.2019	08.10.2019	6,830	175,443	12,364	2,902
**2020/21**	48	02.06.2020	07.05.2021	9,606	99,587	16,924	1,931
**All**				**41,234**	**934,810**	**8 0,191**	**14,442**

**Table 2.** Number of sequences (observations) of each species per year and in total.

**Species**		**2015**	**2016**	**2017**	**2018**	**2019**	**2020 - 2021**	**Total**
**Wild**								
**European roe deer**	*Capreoluscapreolus*	463	520	150	156	616	643	**2,548**
**Wild boar**	*Susscrofa*	267	286	138	169	677	354	**1,891**
**Red fox**	*Vulpesvulpes*	92	100	1	5	106	72	**376**
**Gray wolf**	*Canislupus*	14	40	5	19	37	5	**120**
**Stone marten**	*Martesfoina*	4	5	0	1	6	0	**16**
**Iberian ibex**	*Caprapyrenaica*	0	3	0	2	9	38	**52**
**Common genet**	*Genettagenetta*	0	2	0	0	5	3	**10**
**Red deer**	*Cervuselaphus*	0	0	0	5	4	0	**9**
**Eurasian badger**	*Melesmeles*	1	0	0	0	3	5	**9**
**European rabbit**	*Oryctolaguscuniculus*	2	6	0	0	0	0	**8**
**Domestic**								
**Domestic cattle**	*Bostaurus*	1,297	1,369	327	460	764	355	**4,572**
**Domestic horse**	*Equuscaballus*	660	1,320	1,088	452	617	417	**4,554**
**Domestic dog**	*Canislupusfamiliaris*	38	68	1	2	14	21	**144**
**Domestic sheep**	*Ovisaries*	3	18	4	1	0	0	**26**
**Domestic goat**	*Caprahircus*	1	5	1	7	9	0	**23**
**Domestic cat**	*Feliscatus*	0	0	0	1	1	4	**6**
**Other**								
**Birds**	*Aves*	8	13	0	3	8	11	**43**
**Rodents**	*Rodentia*	0	5	0	0	25	3	**33**
**Lizards**	*Reptilia*	0	1	0	0	1	0	**2**

**Table 3.** Density estimates of the six most common species in 2019. n = sample size (total number of first trigger images), n_model_ = number of first trigger images included in the distance sampling model after truncation, \begin{varwidth}{50in}\begin{equation*}
            \varepsilon
        \end{equation*}\end{varwidth} = mean encounter rate, a = activity level / availability factor, p = detection probability, D = density (individuals per km²), CV = coefficient of variation.

**Species**		**n**	**n_model_**	***e* [CV**]	**a [CV**]	**p [CV**]	**D [CV**]
**Gray wolf**	*Canislupus*	88	77	0.22 [34%]	0.35 [9%]	0.24 [44%]	**0.15 [56**%]
**Red fox**	*Vulpesvulpes*	146	114	0.16 [19%]	0.53 [11%]	0.16 [23%]	**0.30 [32**%]
**European roe deer**	*Capreoluscapreolus*	1,109	672	0.83 [16%]	0.44 [4%]	0.06 [8%]	**5.88 [18**%]
**Wild boar**	*Susscrofa*	2,057	1,754	1.01 [21%]	0.44 [3%]	0.25 [5%]	**2.59 [22**%]
**Domestic horse**	*Equuscaballus*	6,801	2,610	6.19 [25%]	0.55 [2%]	0.11 [3%]	**10.89 [25**%]
**Domestic cattle**	*Bostaurus*	6,321	1,046	5.19 [28%]	0.36 [2%]	0.13 [7%]	**23.39 [29**%]

## References

[B8028735] Akaike Hirotugu, Petrov B. N., Csaki F. (1973). International symposium on information theory.

[B8247254] Bosch Jaime, Peris Salvador, Fonseca Carlos, Martinez Marta, Torre Ana De la, Iglesias Irene, Muñoz Maria J. (2012). Distribution, abundance and density of the wild boar on the Iberian peninsula, based on the CORINE program and hunting statistics. Folia Zoologica.

[B8002302] Buckland S. T., Anderson D. R., Burnham K. P., Laake J. L., Borchers D. L., Thomas L. (2004). Advanced distance sampling.

[B8002213] Burton A. Cole, Neilson Eric, Moreira Dario, Ladle Andrew, Steenweg Robin, Fisher Jason T., Bayne Erin, Boutin Stan (2015). Review: Wildlife camera trapping: a review and recommendations for linking surveys to ecological processes. Journal of Applied Ecology.

[B8028911] Campos Rafael (2018). Survival and population dynamics of the Iberian wolf in Portugal. Dissertação de Mestrado. Universidade do Porto. Biodiversidade, Genética e Evolução.

[B8028929] Team Camtrap DP Development Camera Trap Data Package. https://github.com/tdwg/camtrap-dp.

[B8002226] Caravaggi Anthony, Banks Peter B., Burton A Cole, Finlay Caroline M. V., Haswell Peter M., Hayward Matt W., Rowcliffe Marcus J., Wood Mike D. (2017). A review of camera trapping for conservation behaviour research. Remote Sensing in Ecology and Conservation.

[B8120704] Casaer Jim, Milotic Tanja, Liefting Yorick, Desmet Peter, Jansen Patrick (2019). Agouti: A platform for processing and archiving of camera trap images. Biodiversity Information Science and Standards.

[B8028937] Group Darwin Core Task Darwin Core. Biodiversity Information Standards (TDWG). http://www.tdwg.org/standards/450.

[B8028748] Dorfman R. (1938). A note on the δ-Method for finding variance formulae. The Biometric Bulletin.

[B8028857] Efford Murray G., Dawson Deanna K. (2012). Occupancy in continuous habitat. Ecosphere.

[B8002174] Fewster Rachel M, Buckland Stephen T, Burnham Kenneth P, Borchers David L, Jupp Peter E, Laake Jeffrey L, Thomas Len (2008). Estimating the encounter rate variance in distance sampling.. Biometrics.

[B8321680] Fiske Ian, Chandler Richard (2011). Unmarked: An R package for fitting hierarchical models of wildlife occurrence and abundance. Journal of Statistical Software.

[B8247183] Fonseca Carlos, Migueis David, Fernandes Tony, Carvalho Henrique, Loureiro Armando, Carvalho João, Torres Rita Tinoco (2017). The return of the Iberian wild goat *Caprapyrenaica* to Portugal: From reintroduction to recolonization. Journal for Nature Conservation.

[B8109093] Gabarrón-Galeote Miguel A., Trigalet Sylvain, Wesemael Bas van (2015). Soil organic carbon evolution after land abandonment along a precipitation gradient in southern Spain. Agriculture, Ecosystems & Environment.

[B8028846] Gaston Kevin J., Blackburn Tim M., Greenwood Jeremy J. D., Gregory Richard D., Quinn Rachel M., Lawton John H. (2000). Abundance-occupancy relationships. Journal of Applied Ecology.

[B8002273] Gilbert Neil A., Clare John D. J., Stenglein Jennifer L., Zuckerberg Benjamin (2020). Abundance estimation of unmarked animals based on camera‐trap data. Conservation Biology.

[B8109111] Hatna Erez, Bakker Martha M. (2011). Abandonment and expansion of arable land in Europe. Ecosystems.

[B8002165] Howe Eric J., Buckland Stephen T., Després‐Einspenner Marie‐Lyne, Kühl Hjalmar S. (2017). Distance sampling with camera traps. Methods in Ecology and Evolution.

[B8247323] (INE) Instituto Nacional de Estatística (2019). Efetivo bovino habitual (N.º) por Localização geográfica (NUTS - 2013), Categoria (efetivo bovino) e Sistema de produção; Decenal - INE, Recenseamento agrícola - 2019.

[B8002282] Karanth K. Ullas (1995). Estimating tiger *Pantheratigris* populations from camera-trap data using capture—recapture models. Biological Conservation.

[B8028696] MacKenzie Darryl I., Nichols James D., Lachman Gideon B., Droege Sam, Andrew Royle J., Langtimm Catherine A. (2002). Estimating site occupancy rates when detection probabilities are less than one. Ecology.

[B8085649] Moço Gisela, Guerreiro Margarida, Ferreira Ana Filipa, Rebelo António, Loureiro Armando, Petrucci-Fonseca Francisco, Pérez Jesús Ma (2006). The ibex *Caprapyrenaica* returns to its former Portuguese range. Oryx.

[B8109134] Navarro Laetitia M., Pereira Henrique M., Pereira Henrique M., Navarro Laetitia M. (2015). Rewilding European Landscapes.

[B8109102] Nunes A. N., Coelho C. O. A., de Almeida A. C., Figueiredo A. (2010). Soil erosion and hydrological response to land abandonment in a central inland area of Portugal. Land Degradation & Development.

[B8002239] O’Connell Allan F., Nichols James D., Karanth K. Ullas (2011). Camera traps in animal ecology.

[B8028892] Pimenta V., Barroso L. ;, Álvares F., Ferrão da Costa G. ;, Moreira L., Nascimento J., Petrucci-Fonseca F., Roque S., Santos E. (2005). Situação Populacional do Lobo em Portugal: resultados do Censo Nacional 2002/2003.

[B8247266] Pittiglio Claudia, Khomenko Sergei, Beltran-Alcrudo Daniel (2018). Wild boar mapping using population-density statistics: From polygons to high resolution raster maps. PLOS One.

[B8002186] Rodrigues Patrícia (2010). Landscape changes in Castro Laboreiro: from farmland abandonment to forest regeneration. Dissertation (M.Sc.).

[B8028815] Rovero F., Tobler M., Sanderson J., Eymann J., Degreef J., Häuser Ch., Monje JC., Samyn Y., Vandenspiegel D. (2010). Manual on field recording techniques and protocols for all taxa biodiversity inventories and monitoring..

[B8002252] Rowcliffe J. M., Carbone C. (2008). Surveys using camera traps: are we looking to a brighter future?. Animal Conservation.

[B8028686] Rowcliffe J. Marcus, Kays Roland, Kranstauber Bart, Carbone Chris, Jansen Patrick A. (2014). Quantifying levels of animal activity using camera trap data. Methods in Ecology and Evolution.

[B8085663] Santos João Pedro Valente e, Carvalho João Luís, Torres Rita Tinoco, Gortázar Christian, Fonseca Carlos, Biologists International Union of Game (2011). Red deer in northeastern Portugal: monitoring trends in a transboundary population. Conference: XXXth IUGB (International Union of Game Biologists) Congress and PERDIX XIII.

[B8162560] Hídricos SNIRH - Sistema Nacional de Informação de Recursos Portelinha (01H/02G) - Precipitação anual (mm). http://snirh.apambiente.pt.

[B8028707] Thomas Len, Buckland Stephen T., Rexstad Eric A., Laake Jeff L., Strindberg Samantha, Hedley Sharon L., Bishop Jon R. B., Marques Tiago A., Burnham Kenneth P. (2010). Distance software: design and analysis of distance sampling surveys for estimating population size. Journal of Applied Ecology.

[B8247244] Torres Rita Tinoco, Miranda João, Carvalho João, Fonseca Carlos (2015). Expansion and current status of roe deer (*Capreoluscapreolus*) at the edge of its distribution in Portugal. Annales Zoologici Fennici.

[B8328449] Valente Ana M., Fonseca Carlos, Marques Tiago A., Santos João P., Rodrigues Rogério, Torres Rita Tinoco (2014). Living on the edge: Roe deer (*Capreoluscapreolus*) density in the margins of its geographical range. PLOS One.

[B8002195] van der Zanden Emma H., Carvalho-Ribeiro Sónia M., Verburg Peter H. (2018). Abandonment landscapes: user attitudes, alternative futures and land management in Castro Laboreiro, Portugal. Regional Environmental Change.

[B8343452] Zuleger A, Perino A, Wolf F, Pereira H, Wheeler H (2023). Long-term monitoring of mammal communities in the Peneda-Gerês National Park using camera trap data. Sampling event dataset.

