# Supplementary material for: Long-term monitoring of mammal communities in the Peneda-Gerês National Park using camera-trap data
Source: Biodivers Data J. 2023 Apr 20;11:e99588. doi: 10.3897/BDJ.11.e99588 (PMC10848441; doi:10.3897/BDJ.11.e99588)
Supplement: Supplementary material 1 — Occupancy and detection probabilities per species and year [file bdj-11-e99588-s001.pdf]

Table A1 Occupancy and detection probabilities and standard errors (SE) per species and year.

Species	Year	Occupancy	Occupancy SE	Detection	Detection SE
Eurasian badger	2015	0.69	0.46	0.00	0.00
Eurasian badger	2016	NA	NA	NA	NA
Eurasian badger	2017	NA	NA	NA	NA
Eurasian badger	2018	NA	NA	NA	NA
Eurasian badger	2019	0.98	0.50	0.00	0.00
Eurasian badger	2020	0.66	0.46	0.00	0.00
Domestic cattle	2015	0.62	0.07	0.13	0.01
Domestic cattle	2016	0.68	0.07	0.10	0.00
Domestic cattle	2017	0.72	0.07	0.05	0.00
Domestic cattle	2018	0.47	0.06	0.06	0.00
Domestic cattle	2019	0.46	0.08	0.08	0.01
Domestic cattle	2020	0.45	0.08	0.04	0.00
Gray wolf	2015	0.25	0.09	0.02	0.01
Gray wolf	2016	0.15	0.05	0.04	0.01
Gray wolf	2017	1.00	0.47	0.00	0.00
Gray wolf	2018	0.18	0.07	0.01	0.00
Gray wolf	2019	0.21	0.07	0.02	0.00
Gray wolf	2020	NA	NA	NA	NA
Domestic horse	2015	0.62	0.07	0.09	0.01
Domestic horse	2016	0.71	0.06	0.10	0.00
Domestic horse	2017	0.73	0.06	0.09	0.00
Domestic horse	2018	0.64	0.07	0.05	0.00
Domestic horse	2019	0.49	0.07	0.07	0.00
Domestic horse	2020	0.46	0.07	0.03	0.00
Red fox	2015	0.31	0.06	0.06	0.01
Red fox	2016	0.56	0.07	0.03	0.00
Red fox	2017	0.98	0.50	0.00	0.00
Red fox	2018	0.05	0.04	0.01	0.01
Red fox	2019	0.46	0.09	0.03	0.00
Red fox	2020	0.26	0.07	0.02	0.00
European roe deer	2015	0.95	0.04	0.09	0.00
European roe deer	2016	0.91	0.05	0.08	0.00
European roe deer	2017	0.72	0.07	0.03	0.00
European roe deer	2018	0.72	0.07	0.03	0.00
European roe deer	2019	1.00	0.50	0.07	0.00
European roe deer	2020	0.82	0.05	0.04	0.00
Wild boar	2015	0.64	0.07	0.07	0.01
Wild boar	2016	0.65	0.07	0.05	0.00
Wild boar	2017	0.48	0.07	0.03	0.00
Wild boar	2018	0.64	0.07	0.04	0.00
Wild boar	2019	0.92	0.04	0.08	0.00
Wild boar	2020	0.70	0.07	0.04	0.00
Common genet	2015	NA	NA	NA	NA
Common genet	2016	0.79	0.44	0.00	0.00
Common genet	2017	NA	NA	NA	NA
Common genet	2018	NA	NA	NA	NA
Common genet	2019	0.05	0.04	0.04	0.02
Common genet	2020	0.02	0.05	0.02	0.01
Iberian ibex	2015	NA	NA	NA	NA
Iberian ibex	2016	0.07	0.11	0.01	0.01
Iberian ibex	2017	NA	NA	NA	NA
Iberian ibex	2018	0.03	0.05	0.01	0.01
Iberian ibex	2019	0.02	0.03	0.05	0.02
Iberian ibex	2020	0.05	0.03	0.04	0.01
Red deer	2015	NA	NA	NA	NA
Red deer	2016	NA	NA	NA	NA
Red deer	2017	NA	NA	NA	NA
Red deer	2018	0.02	0.06	0.02	0.01
Red deer	2019	0.03	0.04	0.02	0.01
Red deer	2020	NA	NA	NA	NA
