# Supplementary figures and images for: Long-term monitoring of mammal communities in the Peneda-Gerês National Park using camera-trap data
Source: Biodivers Data J. 2023 Apr 20;11:e99588. doi: 10.3897/BDJ.11.e99588 (PMC10848441; doi:10.3897/BDJ.11.e99588)

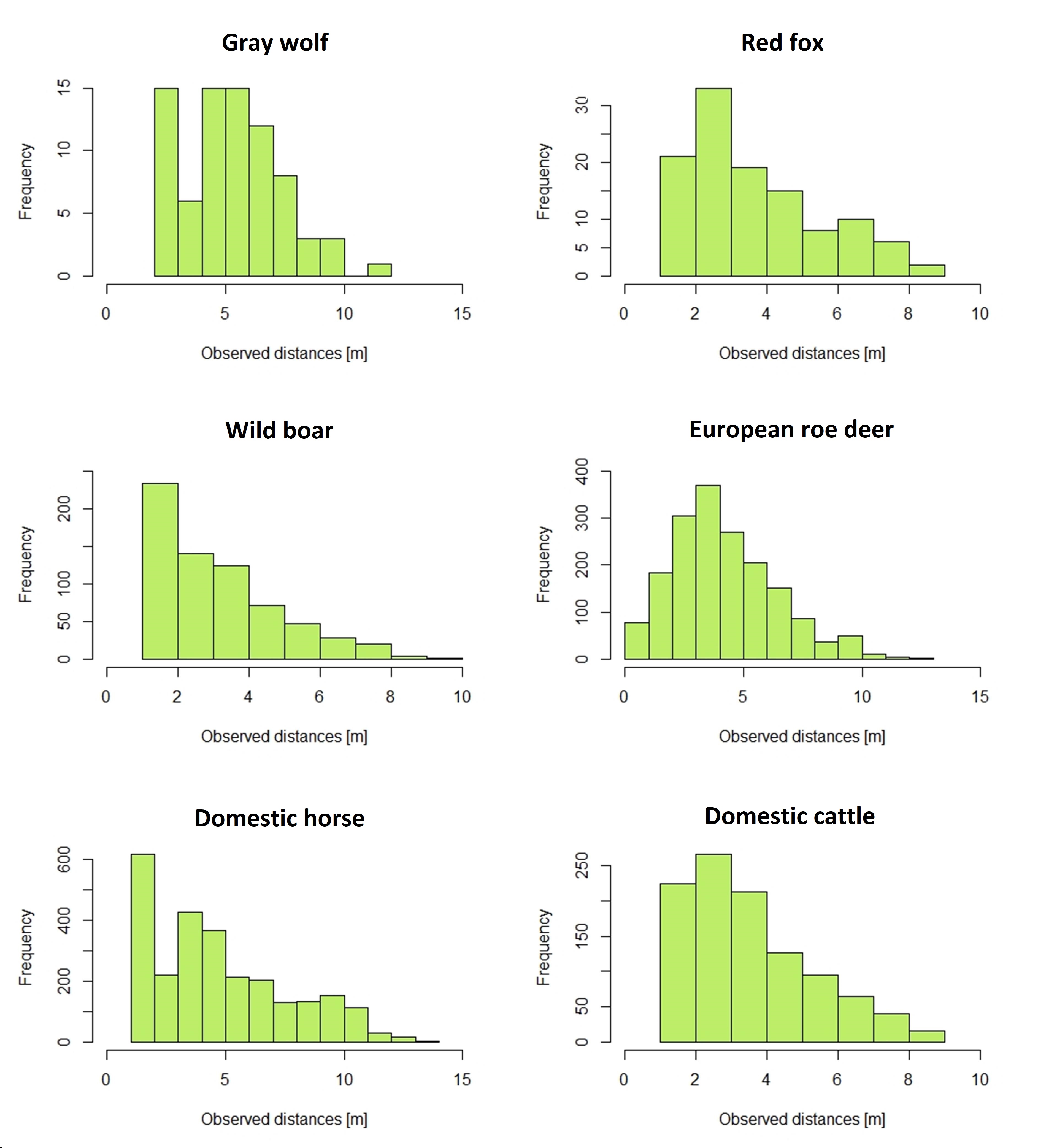

Supplement: Supplementary material 2 — Observation distances [file bdj-11-e99588-s002.jpg]
