# Supplementary material for: Long-term monitoring of mammal communities in the Peneda-Gerês National Park using camera-trap data
Source: Biodivers Data J. 2023 Apr 20;11:e99588. doi: 10.3897/BDJ.11.e99588 (PMC10848441; doi:10.3897/BDJ.11.e99588)
Supplement: Supplementary material 3 — Distance-sampling models [file bdj-11-e99588-s003.pdf]

Table A2 Models fitted with Distance and weighted by AIC for the total survey area. # Adj params = number of cosine adjustment parameters, # params = number of parameters in the model, w = AIC weight, P = detection probability, P(w) = weighted detection probability, CV = Coefficient of variation, CV(w) = weighted coefficient of variation, GOF Chi-p = Chi-Square Goodness of Fit Test. Models that produced errors are not included.

Species	Name	Key function	# Adj params	# params	AIC	Delta AIC	w	P	P(w)	CV	CV(w)	GOF Chi-p
**Gray wolf**	hn_cos 2_10m	Half-normal	2	3	307.86	0.00	0.27	0.09	0.21	0.49	0.44	0.41
	hn_cos0_10m	Half-normal	0	1	308.07	-0.21	0.25	0.26		0.16		0.30
	hr_cos 2_10m	Hazard-rate	2	4	308.90	-1.04	0.16	0.10		0.92		0.38
	hr_cos 1_10m	Hazard-rate	1	3	309.61	-1.75	0.11	0.23		0.39		0.26
	hn_cos 1_10m	Half-normal	1	2	309.83	-1.97	0.10	0.34		0.58		0.21
	hr_cos0_10m	Hazard-rate	0	2	309.90	-2.04	0.10	0.45		0.13		0.14
**Red fox**	hr_cos0_9m	Hazard-rate	0	2	432.30	0.00	0.31	0.19	0.16	0.18	0.23	0.58
	hn_cos1_9m	Half-normal	1	2	432.31	-0.01	0.31	0.12		0.18		0.58
	hn_cos2_9m	Half-normal	2	3	433.73	-1.43	0.15	0.14		0.28		0.52
	hr_cos 1_9m	Hazard-rate	1	3	434.30	-2.00	0.12	0.19		0.23		0.44
	hn_cos0_9m	Half-normal	0	1	435.65	-3.35	0.06	0.19		0.11		0.11
	hr_cos 2_9m	Hazard-rate	2	4	435.96	-3.66	0.05	0.15		0.97		0.33
**European roe deer**	hn_cos 2_10m	Half-normal	2	3	2334.70	0.00	0.98	0.06	0.06	0.08	0.08	0.00
	hn_cos 1_10m	Half-normal	1	2	2342.80	-8.09	0.02	0.07		0.05		0.00
	hr_cos0_10m	Hazard-rate	0	2	2357.72	-23.02	0.00	0.08		0.09		0.00
	hr_cos1_10m	Hazard-rate	1	3	2359.72	-25.02	0.00	0.08		0.09		0.00
	hn_cos0_10m	Half-normal	0	1	2394.11	-59.41	0.00	0.11		0.04		0.00
**Wild boar**	hr_cos0_10m	Hazard-rate	0	2	7321.51	0.00	0.71	0.28	0.28	0.03	0.05	0.01
	hr_cos 1_10m	Hazard-rate	1	3	7323.51	-2.00	0.26	0.28		0.09		0.00
	hn_cos 2_10m	Half-normal	2	3	7328.20	-6.68	0.03	0.25		0.08		0.00
	hn_cos0_10m	Half-normal	0	1	7336.09	-14.58	0.00	0.22		0.03		0.00
	hn_cos1_10m	Half-normal	1	2	7338.09	-16.58	0.00	0.22		0.06		0.00
**Domestic horse**	hn_cos1_12m	Half-normal	1	2	11724.61	0	0.54	0.11	0.11	0.03	0.03	0.00
	hn_cos2_12m	Half-normal	2	3	11724.97	-0.36	0.46	0.11		0.04		0.00
	hr_cos2_12m	Hazard-rate	2	4	11744.82	-20.21	0.00	0.11		0.04		0.00
	hn_cos0_12m	Half-normal	0	1	12082.86	-358.25	0.00	0.20		0.02		0.00
**Domestic cattle**	hn_cos1_9m	Half-normal	1	2	3851.89	0.00	0.53	0.12	0.13	0.06	0.07	0.60
	hn_cos2_9m	Half-normal	2	3	3852.16	-0.27	0.46	0.13		0.09		0.75
	hr_cos0_9m	Hazard-rate	0	2	3860.33	-8.44	0.01	0.18		0.06		0.05
	hr_cos1_9m	Hazard-rate	1	3	3862.33	-10.44	0.00	0.18		0.08		0.03
	hn_cos0_9m	Half-normal	0	1	3879.69	-27.8	0.00	0.17		0.04		0.00
